# Leech Bite: A Rare Cause of Postmenopausal Vaginal Bleeding

**Published:** 2012-06-30

**Authors:** M Hasanzadeh Mofrad, R Shafiei, S Bolandi, M Najjari, G R Hatam

**Affiliations:** 1 Department of Obstetrics and Gynecology, Ghaem Hospital, Gynecological Health Research Center, Mashhad University of Medical Sciences, Mashhad, Iran; 2 Department of Parasitology and Mycology, School of Medicine, Shiraz University of Medical Sciences, Shiraz, Iran; 3 Diagnostic Laboratory Sciences and Technology Research Center, Shiraz University of Medical Sciences, Shiraz, Iran

**Keywords:** Leech, Bite, Postmenopausal vaginal bleeding, Iran

Dear Editor,

Postmenopausal vaginal bleeding has various causes. Differential diagnoses include exogenous estrogens, atrophic endometritis/vaginitis, endometrial or cervical cancer and polyps, uterine sarcoma, urethral caruncles and trauma.[1] Therefore, it is necessary to rule out the pathological etiology of bleeding in postmenopausal women.

The leech is a member of the phylum Annelida of the class Hirudinea and a blood-sucking aquatic ectoparasite. Its size varies from about 5 mm to nearly 45 cm. Leeches are found in rivers and ponds in tropical and subtropical regions. The leech has an oral sucker as a mouth, and a caudal sucker for movement.[2][3]

Leech bite in various human body sites such as the nose, pharynx, larynx, esophagus, rectum, and bladder has been reported sporadically in the literature.[4] It is commonly found in children after bathing in the river. However, leech bite in the vagina is an uncommon problem. It is a very rare cause of vaginal bleeding but it should be kept in mind, especially in people who live in poor hygienic conditions.[5]

A 79-year-old woman, with a postmenopausal period of 29 years, referred to the Gynecology and Oncology Department of Ghaem Hospital in Mashhad, Northeast Iran with vaginal bleeding in order to rule out cancer. She had had such complaints since three months before the referral. She suffered from continuous moderate to severe vaginal bleeding and moderate vertigo. The patient did not complain of any other symptoms. She did not live in a hygienic and healthy environment, and she used to wash her external genitalia with infected water. The only specific event worth mentioning in her history was that she had undergone vaginal hysterectomy because of a prolapsed uterus five years earlier.

**Fig. 1:**
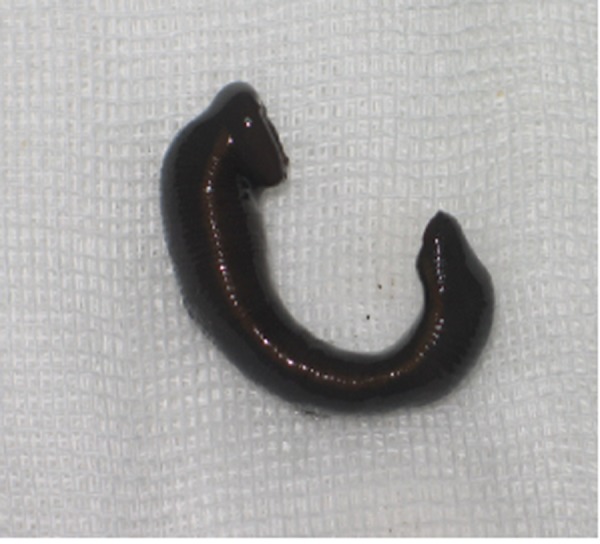
The 5-centimeter leech that was stuck to the mucosa of the distal segment of the vagina.

Upon physical examination, she seemed pale and her vital signs were as follows: Heart rate=110 beat/min, blood pressure=80/60 mm hg, temperature=36.8°C, and respiratory rate=28/min. During gynecological examination, no abnormality was noticed in the vulva. Atrophic bloody mucosa was seen upon vaginal inspection with a speculum, and one leech, 5 cm in length, was stuck to the mucosa of the distal segment of the vagina (Figure 1). Then the vagina was washed with physiological normal saline solution to clean the bleeding point. However, the leech was not removed. The laboratory tests showed: hemoglobin=5.9 mg/dL, hematocrit=19%, platelet count=149000, prothrombin time=14, partial thromboplastin time=40 and INR=1.

The patient was then scheduled for surgery. In the operating room, after general anesthesia, the vagina was explored with two valves. Because the leech was completely stuck to the mucosa, we injected lidocaine directly to the body of the leech to make it paralyzed and relaxed. Subsequently, the leech was removed, and vaginal bleeding stopped. In order to stabilize the patient’s vital signs, 2 units of packed cells were transfused. In the follow-up visits, the patient was well and no other pathological finding was found.

The leech and its uses and complications have a long lasting history in medicine. It was used by Greek and Moorish physicians for leech phlebotomy.[1] In case it enters the vagina, it can cause vaginal bleeding.[5][6][7] In a young girl with vaginal bleeding after swimming in rivers or ponds, physicians are advised to consider the leech as a cause of bleeding.[5]

By searching the Medline database, we found that leech bites in the vagina are an uncommon problem that caused vaginal bleeding.[5][6][7] However, leech bites should be considered in patients who present with vaginal bleeding and live in damp areas [5] as well as those elderly women with vaginal bleeding who have bad hygienic facilities similar to the case we presented.

The continuous bleeding after a leech bite results from the action of substances in the leech's saliva which is left in the bitten part, including histamine-like vasodilators, hirudin (a potent antithrombin), hyaluronidase, and calin (a platelet aggregation inhibitor).[8] Bleeding from a leech bite wound can persist for a mean of 10 hours and last for as long as 7 days.[9]

The treatment of a leech bite in the vagina can be supportive. If the patient's coagulation profile is normal, only blood transfusion is needed to replace the amount of blood loss. The leech should not be forcibly removed because its jaws may remain in the wound, causing continuous bleeding and infection.[10] Patients who develop complications need extra medical care.

Since this is the first report of vaginal bleeding caused by leech bites in our reported area, physicians should consider leeches as one of the causes of vaginal bleeding even if it happens during the postmenopausal period. The correct early diagnosis would help the patient greatly and reduce morbidity.
